# The crystal structure of human forkhead box N1 in complex with DNA reveals the structural basis for forkhead box family specificity

**DOI:** 10.1074/jbc.RA119.010365

**Published:** 2019-12-30

**Authors:** Joseph A. Newman, Hazel Aitkenhead, Angeline E. Gavard, Ioanna A. Rota, Adam E. Handel, Georg A. Hollander, Opher Gileadi

**Affiliations:** ‡ Structural Genomics Consortium, University of Oxford, Oxford OX3 7DQ, United Kingdom; § Department of Paediatrics and the Weatherall Institute of Molecular Medicine, University of Oxford, Oxford OX3 9DS, United Kingdom; ¶ Nuffield Department of Clinical Neurosciences, University of Oxford, Oxford OX3 9DU, United Kingdom; ‖ Paediatric Immunology, Department of Biomedicine, University of Basel and University Children's Hospital Basel, 4056 Basel, Switzerland

**Keywords:** DNA–protein interaction, immunodeficiency, transcription factor, crystal structure, gene regulation, forkhead box N1, thymus

## Abstract

Forkhead box N1 (FOXN1) is a member of the forkhead box family of transcription factors and plays an important role in thymic epithelial cell differentiation and development. *FOXN1* mutations in humans and mice give rise to the “nude” phenotype, which is marked by athymia. FOXN1 belongs to a subset of the FOX family that recognizes an alternative forkhead-like (FHL) consensus sequence (GACGC) that is different from the more widely recognized forkhead (FKH) sequence RYAAAYA (where R is purine, and Y is pyrimidine). Here, we present the FOXN1 structure in complex with DNA containing an FHL motif at 1.6 Å resolution, in which the DNA sequence is recognized by a mixture of direct and water-mediated contacts provided by residues in an α-helix inserted in the DNA major groove (the recognition helix). Comparisons with the structure of other FOX family members revealed that the FKH and FHL DNA sequences are bound in two distinct modes, with partially different registers for the protein DNA contacts. We identified a single alternative rotamer within the recognition helix itself as an important determinant of DNA specificity and found protein sequence features in the recognition helix that could be used to predict the specificity of other FOX family members. Finally, we demonstrate that the C-terminal region of FOXN1 is required for high-affinity DNA binding and that FOXN1 has a significantly reduced affinity for DNA that contains 5′-methylcytosine, which may have implications for the role of FOXN1 in thymic involution.

## Introduction

The FOX family of transcription factors is one of the largest in humans, with 50 members identified to date (1). FOX family proteins play important roles in various cellular processes including the regulation of cell differentiation, proliferation, metabolism, and senescence. FOX family proteins share a ∼100-amino acid DNA-binding domain (forkhead or FH domain), which is widely conserved throughout evolution from humans to yeast and has been used to classify FOX family proteins into 19 subfamilies (denoted FOXA–FOXS) (2). FOXN1 is a FOX family transcription factor that primarily functions as an activator regulating the development of epithelial cells in skin and thymus. The lack of functional FOXN1 protein expression in humans as well as in other vertebrates causes congenital alopecia universalis, nail dystrophy, and athymia—the so-called “nude” phenotype (3, 4). The latter is due to an arrest in thymic epithelial cell (TEC)^3^ differentiation beyond a progenitor cell state and causes severe T-cell immunodeficiency. The biological targets of FOXN1 in adult TEC were recently identified in a genome-wide study (5). In addition to the control of genes involved in TEC differentiation and proliferation, this analysis also demonstrated that FOXN1 controls, among other things, the expression of genes involved in antigen presentation and processing including peptidases, proteasome subunits, and protein transporters.

FOXN1 is a 648-amino acid protein with the FH domain located centrally between amino acids 270 and 367. No other recognizable domains have been identified, although the N-terminal region has been implicated in thymic epithelial cell differentiation because mice lacking the first 154 amino acids displayed a milder thymus phenotype in comparison the full nude phenotype but maintained a normal coat (6). Similarly, an acidic cluster of amino acids within the C-terminal 175-amino acid terminus has been identified to contain a transcriptional activation domain (7).

FH domains comprise a subclass of the much larger and more diverse winged helix superfamily and comprise three α helices, which form a helix–turn–helix core, followed by a three-stranded mixed β-sheet. The “wings” for which the fold is named are generally less well-conserved across family members. The first wing (wing 1) is formed by an extended loop between strands β2 and β3, whereas the second wing (wing 2) constitutes the residues immediately following from strand β3. Early structural studies on human FOXA3/HNF-3 established a conserved mode of DNA recognition whereby the third α-helix (α3 or “recognition helix”) is inserted deep within the major groove of the DNA and provides direct and water-mediated sequence-specific contacts to the DNA bases that facilitate DNA recognition (8). The recognition motif for the majority of forkhead domains has the seven-base FKH consensus pattern RYAAAYA. In contrast, FOXN1 recognizes an alternate 5-bp DNA motif, GACGC. This alternate motif is also bound by a subset of FOX proteins, designated FHL (named after the *Saccharomyces cerevisiae Fhl1* gene, the first family member to show this binding property). The recognition of the alternate FHL motif GACGC by FOXN1 and a subset of forkhead proteins is particularly puzzling because the sequences of the core DNA contacting residues within the recognition helix α3 are strictly conserved even across family members with different specificities. A recent analysis of the evolution of these alternate specificities within the FOX family indicates that the alternate specificity has evolved independently in three different phylogenetic lineages (9). Moreover, some bi-specific proteins have also been identified that are able to bind with high affinity to both motifs. Understanding the basis of recognition of divergent DNA sequences will require molecular structures of FH domain(s) bound to the alternate motif.

To date, a number of structures of FOX family proteins have been determined in complex with FKH DNA sequences, including human FOXA2 (10), FOXK1 (11), FOXO1 (12), FOXO3 (13), FOXO4 (14), FOXP2 (15), and FOXM1 (16). All structures show the same basic arrangement of direct and water-mediated sequence-specific contacts provided by residues in α3, underlying the recognition of the FKH consensus sequence RYAAAYA. In contrast, the features that mediate recognition of the alternate FHL motif are less well-understood. A recent study on the bi-specific FOX family member FOXN3 bound to both FKH and FHL sequences (17) showed that the two DNA sequences are bound in a distinctly different conformation, allowing the same amino acids to make contact with different DNA bases. However, the moderate resolution of these structures (2.6 Å for FKH and 2.7 Å for FHL) precluded a detailed analysis of the water-mediated contacts and hydrogen-bonding networks that are a general feature of specific DNA recognition by transcription factors. In this study, we describe the crystal structures of human FOXN1 both alone and in complex with DNA at 2.7 and 1.6 Å resolution, respectively. This is the first structure of any FHL-specific FOX family member bound to a noncanonical FHL motif GACGC. Detailed analysis of the structure reveals a distinct mechanism used by FOXN1 to recognize its specific DNA motif. Comparisons with previous FOX family DNA complexes show that although the conformation of the recognition helix remains largely unchanged, a single alternate rotamer adopted by a conserved asparagine in the recognition permits the binding of DNA in an alternative manner, providing a different register for base-specific contacts. We identify amino acid sequences immediately upstream of the recognition helix that appear to be a requirement for FHL binding and may serve as predictive features for FHL binding in other FOX family members.

## Results and discussion

### Structure of human FOXN1

**Figure 1.**
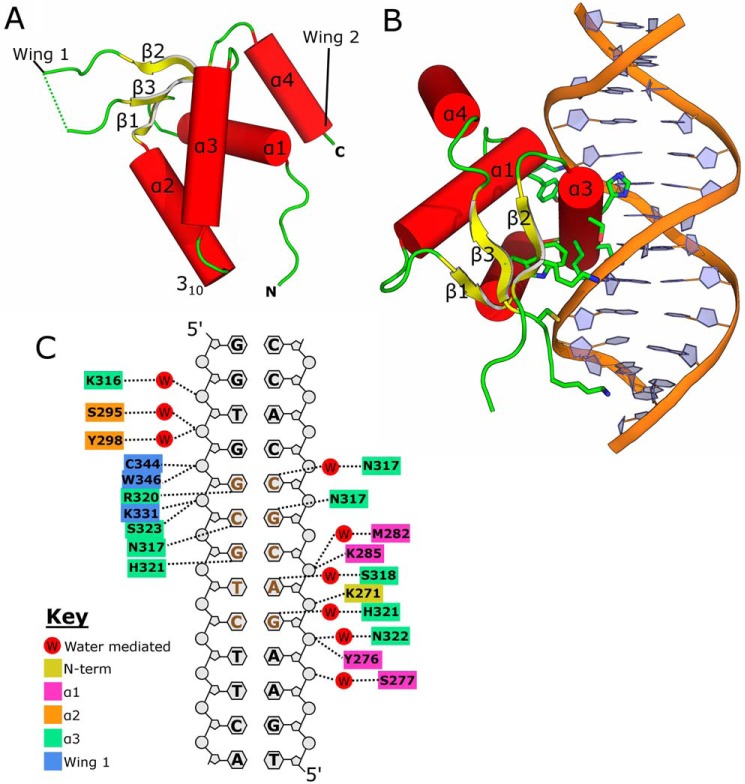
**Structures of FOXN1 and the FOXN1 DNA complex.** *A*, overall structure of FOXN1 with secondary structure elements labeled. *B*, structure of the FOXN1 DNA complex with key interacting residues shown in *stick* format. *C*, schematic view of the FOXN1 DNA interaction with polar contacts marked and the FHL motif highlighted in *brown*.

The structure of the forkhead domain (residues 270–366) of human FOXN1 was determined in the presence and absence of DNA at 1.6 and 2.7 Å resolution, respectively. The electron density was of overall good quality except for a seven-residue internal loop and the final five residues at the C terminus, which are not visible in the electron density maps, presumably because of disorder. A summary of the data collection and refinement statistics are shown in Table 1. Significant conformational changes of FOXN1 were not apparent upon the molecule's binding to DNA (RMSD of 0.7 Å over 84 residues). The overall structure of FOXN1 is also very similar to a number of other FH domain family proteins such as FOXM1 (16), FOXP2 (15), and FOXK1 (11) (∼1.1 Å RMSD over ∼80 residues), despite only modest pairwise sequence identities of ∼40%. The most prominent differences between the various structures lie in the sequence, length, and conformation of the wings. Wing 1 of FOXN1 constitutes a relatively long loop that is partially disordered at one end, regardless of whether the molecule is unbound or complexed to DNA (Fig. 1*A*). The wing 2 region forms an additional α-helix (α4), which packs against the rest of the helical core and ends with a stretch of positively charged residues that also remain disordered in electron density maps. Another feature that varies among forkhead structures is the conformation of the loop between helices α2 and α3, which in FOXN1 forms an additional short 3-_10_ helix.

**Table 1:** **Data collection and refinement statistics**

	FOXN1	FOXN1 + DNA
Space group	P 21 21 21	P 1
Cell dimensions (Å)		
*a*	42.1	38.8
*b*	78.2	43.3
*c*	263.5	58.3
Angles (°)		
α	90	90.05
β	90	95.7
γ	90	93.8
Wavelength (Å)	0.976	0.979
Resolution (Å)	43.9–2.70 (2.83–2.70)	34.5–1.61 (1.65–1.61)
*R*_merge_	0.10 (1.32)	0.03 (0.39)
*R*_p.i.m._	0.04 (0.52)	0.03 (0.33)
*I*/σ*I*	11.8 (1.5)	8.1 (1.2)
CC_½_	0.999 (0.876)	0.998 (0.963)
Completeness (%)	99.8 (99.9)	90.7 (53.4)
Multiplicity	7.1 (7.3)	2.1 (1.9)
No. of unique reflections	26,950 (3,240)	44,332 (1,937)
Refinement statistics		
Resolution	43.9–2.70	34.0–1.61
*R*_work_/*R*_free_ (%)	25.11/28.38	19.38/23.94
No. of atoms		
Protein	5307	1,442
Solvent	20	313
DNA		1,054
Average B factors (Å^2^)		
All atoms	84	37
Protein	85	37
Solvent	60	42
DNA		36
Wilson B	67	24.3
RMSD		
Bond lengths (Å)	0.002	0.015
Bond angles (°)	0.455	1.47
Ramachandran plot		
Favored (%)	97.3	94.7
Allowed (%)	2.2	4.09
Protein Data Bank code	5OCN	6EL8

### Recognition of FHL DNA by FOXN1

The consensus sequence for FOXN1 binding has been identified as an invariant stretch of five residues with a consensus sequence 5′-GACGC (5, 18). For the determination of the DNA complex structure, we used a specific 13-nucleotide dsDNA sequence that contained a single copy of this motif flanked by sequences derived from the mouse *Psmb11* promoter (a high confidence target of mouse FOXN1 encoding the proteasome component β5t; TGAA**GACGC**CACC). Comparable with other winged helix superfamily proteins, the third helix of FOXN1 (also referred to as the recognition helix) inserts deep into the major groove of the DNA and provides specific contacts to the nucleotide bases (Fig. 1*B*). Other regions contributing toward DNA binding include the N terminus, the start of the α1 helix, and several residues within and flanking wing 1 that contact the phosphodiester backbone via direct or water-mediated polar interactions (Fig. 1*C*). The overall conformation of the DNA is a modified B form with slight bending toward the protein and concomitant widening of the major groove to accommodate the insertion of α3.

**Figure 2.**
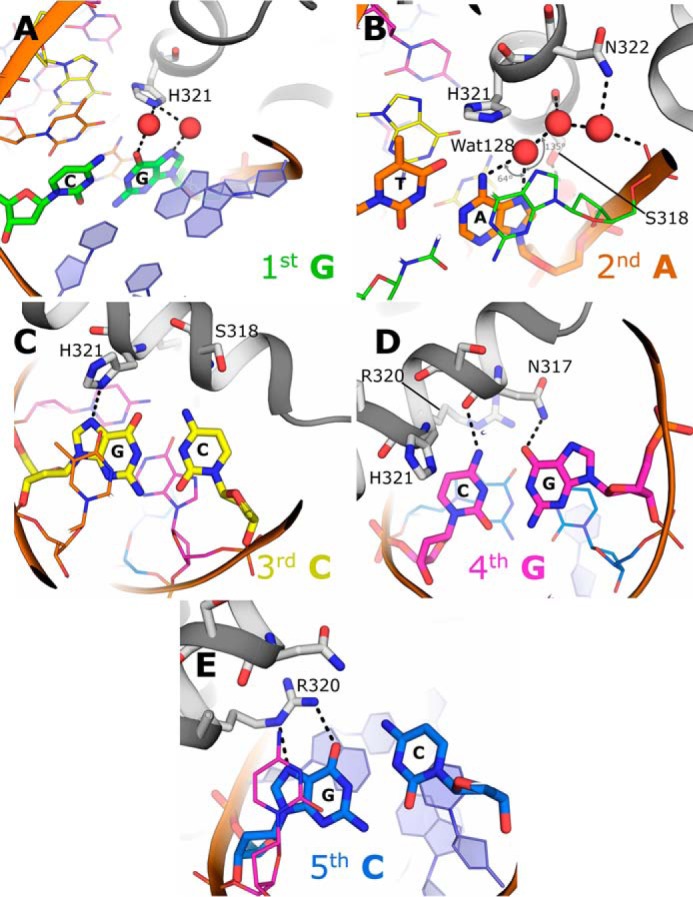
**Details of the recognition of the alternate forkhead motif GACGC by FOXN1.** Key interactions that determine substrate specificity at the first to fifth positions of the motif are shown in *A–E*, respectively. Interacting residues are shown in *stick* format, and water molecules are shown as *red spheres*.

The pattern of polar interactions in the DNA complex structure are summarized schematically in Fig. 1*C*; the details of key protein–base interactions are shown in Fig. 2. FOXN1 recognizes the first base pair (G–C) of the GACGC motif primarily by water-mediated contacts, with two waters within hydrogen-bonding distance of His^321^ making polar contacts to the N7 and O6 groups on the guanine base (Fig. 2*A*). It is not clear how this interaction can underlie a unique discrimination of a G–C pair because the two water molecules could presumably also make hydrogen bonds to an adenine, whereas the nearby histidine (His^321^) could be either a hydrogen bond donor or acceptor depending on the protonation state of the NE2 nitrogen. Thus, some degree of indirect motif recognition may play a role at this position. The second base pair (A–T) is also close to a network of highly coordinated waters, with a single water (at position 128) making a pair of hydrogen bonds to the adenine N7 and N6. Although the pattern of hydrogen-bond donors and acceptors within the water network would permit both donor and acceptor at the N6 position (consistent with the binding of A or G at this position), the angles appear to be more favorable for the water to accept a hydrogen bond from the N6 position (135° *versus* 64°), thus favoring adenine at this position (Fig. 2*B*). Further direct contacts are provided by the side chains of Ser^318^, which is in a position to donate a hydrogen bond to the adenine N7, and His^321^, which makes van der Waals contacts to both the O4 and the (methyl) C7 on the corresponding thymine. The third base pair (C–G) is recognized by FOXN1 via a direct hydrogen bond to the guanine N7 donated by the ND1 of His^321^, together with close van der Waals contacts to the cytosine C5, which, if replaced by a thymine, would cause steric clashes with the side chain of Ser^318^ and the main chain carbonyl of Gly^314^ (Fig. 2*C*). Given that this position forms one of two CpG sites on the motif, we also expect that FOXN1 would, by the same mechanism, be unable to bind to 5-methylcytosine at this position. The fourth base pair (G–C) lies very close to and is directly under the path of the recognition helix, forming two hydrogen bonds with Asn^317^: the N4 of the cytosine donates a hydrogen bond to the main chain carbonyl, and the O6 of the guanine accepts a hydrogen bond from the side chain ND2 (Fig. 2*D*). Like the case at position 3, the C5 of the cytosine at position 4 makes close contacts with the C-terminal half of the recognition helix, and severe steric clashes would occur by the presence of a 5-methyl group (thymine or 5-methycytosine). Finally, the fifth base pair (C–G) is recognized directly by FOXN1 through a pair of hydrogen bonds donated by Arg^320^ to N7 and O6 of the guanine (Fig. 2*E*). This mode of recognition of a guanine by an arginine side chain is common to several other families of transcription factors. The central importance of this interaction is reflected by the fact that a single-point mutation at position Arg^320^ in either human or mice results in a loss of FOXN1 transcriptional activity and consequently in the nude phenotype (19, 20).

### Comparison of FKH and FHL recognition modes

**Figure 3.**
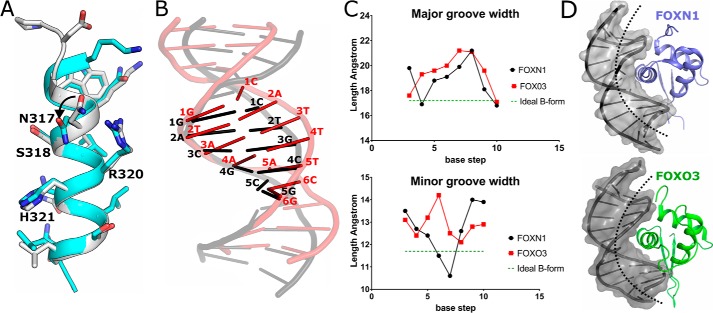
**Comparison of the mode of recognition of the FHK and FHL consensus sequences.** *A*, comparison of the recognition helix of the FHL-bound FOXN1 (shown in *gray*) with the FHK-specific FOXK1 (shown in *cyan*). *B*, comparison of the DNA conformations of the FHL site bound to FOXN1 (*black*) and the FKH site bound to FOXO3 (*red*), DNA molecules are superposed based on the structural alignment of the FOXN1 and FOXO3 proteins, with the respective motifs highlighted. *C*, comparison of the major and minor groove widths for FOXN1 and FOXO3 with values for ideal B-form DNA shown for reference. *D*, space-filling representation of the DNA bound to FOXN1 (*upper panel*) and FOXO3 (*lower panel*). The more prominent bending and greater distance to the recognition helix can be seen in the FOXO3 DNA complex structure.

The structures of several FKH DNA complexes have been determined in various configurations (11, 13, 16). Collectively, these structures share an overall mechanism of base recognition, which is described in more detail in the respective references but will be summarized here to highlight the similarities and differences with DNA recognition by FOXN1. Comparing the FOXN1 mode of recognition detailed above with previous structural studies on FOX family proteins demonstrates both similarities and differences in the DNA–protein interface, which provide insights into how this duality may be achieved. Both the general positioning within the DNA major groove, and the rotamers of key DNA contacting side chains within the recognition helix are generally well-conserved with one notable exception: FOXN1's side chain of Asn^317^ points toward the N terminus of α3, whereas the corresponding side chains of other FOX family structures point in the opposite direction, making extensive contacts with the DNA bases (Fig. 3*A*). Notably, in the DNA-free FOXN1 structure, this same residue is in the same conformation as in the DNA-bound structures of other FOX proteins, and two distinct conformations of the equivalent residue were also observed in the FKH- and FHL-bound FOXN3 structures (17), indicating an induced fit in the FHL DNA complexes.

The most dramatic difference between the FHL and FKH DNA complexes is a change in the register of the DNA: the recognition helix of FOXN1 interacts with a 5-nucleotide FHL sequence GACGC, whereas that helix in other FOX proteins interacts with a 6-bp stretch (RYAAAY). This is achieved by the intercalation of the second (T) nucleotide between the positions occupied by nucleotides 1 and 2 in the FHL motif, without extending the physical length of the DNA motif. Some of the same residues in the recognition helix are used in both binding modes, in particular the binding of His^321^ and Arg^320^ (or their equivalent). This change in register is accompanied by dramatic changes in the conformation of the DNA. Most prominent is the change in the inclination of the bases with respect to the helix axis (up to 24° in the FOXO3 DNA structure compared with near-zero in FOXN1 (Fig. 3*B* and Table S1). This is sufficient to squeeze the 6 bases of the RYAAAY FKH motif into the same helical rise as the 5-base GACGC FHL sequence. Other less striking differences included high positive roll angles, which mainly occur in TA or TG dinucleotide steps in the FKH DNA and significant base pair opening, with generally negative slide and positive roll angles (a full list of DNA geometrical parameters is shown in Table S1). The overall differences in DNA shape are in good agreement with what was observed in the FKH- and FHL-bound FOXN3 structures (17), despite the lengths and sequences of the DNA oligonucleotides being different.

In general, the DNA in the FHL-bound structures are closer to canonical B-form DNA than the FKH DNA structures of other FOX members, although in both cases there is some widening of the major groove (Fig. 3*C*). Taken together, these effects give rise to a more prominent bending of the DNA toward the protein in the FKH complexes, placing the recognition helix generally more distant from the nucleobases (Fig. 3*D*) (∼2 Å further away at the 5′ end of the motif). This increased distance means that residues of the recognition helix that make direct contacts with the bases in the FOXN1 structure make different, water-mediated contacts with bases in the FKH complexes.

**Figure 4.**
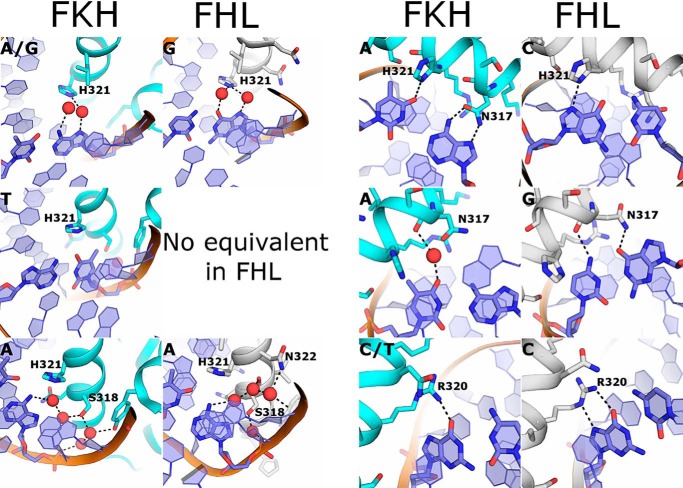
**Comparison of the details of the recognition modes of FKH (*left-hand columns*) and FHL (*right-hand columns*) motif binding.** At each position in the respective motifs, the contacts to the protein that mediate recognition are shown, with the consensus sequence shown throughout in the *top left-hand corner*.

A detailed comparative analysis of the base contacts for each of the two binding modes is shown in Fig. 4. The first base of the FKH motif is either an adenine or guanine, which is contacted by two water molecules coordinated by an adjacent histidine, similar to the interaction of FOXN1 with the first guanine of the FHL sequence; as discussed above, this interaction cannot distinguish between A and G. The second base pair (A–T) in the FKH motif is located between the first and second positions in the FOXN1 structure and does not have an equivalent in that structure. The mode of recognition of the second base of the FKH motif is difficult to determine, although there are possible van der Waals contacts with the thymine methyl. At the third position an invariant adenine base is recognized by a water network that is similar to that used by FOXN1 to recognize adenine at position two of the FHL sequence (Fig. 4, *bottom left panel*). The fourth base pair of the FKH motif is also an invariant A–T and is recognized by a combination of a hydrogen bond donated by the equivalent to His^321^ to the complementary thymine O4 and a bidentate hydrogen bond via the equivalent of Asn^317^, which can recognize uniquely the donor-acceptor pair on the adenine. In the FOXN1 structure, the latter interaction does not occur because of the alternate rotamer of Asn^317^, and His^321^ is hydrogen-bonded to the guanine N7, which occupies approximately the same position as the thymine O4 (Fig. 4, *top right panel*). The fifth base pair in FKL (an invariant A–T) is recognized by a combination of water-mediated contacts to the complementary thymine O4 and hydrophobic interactions with the thymine methyl. In contrast, the equivalent in position in FOXN1 is a guanine-cytosine pair, which is recognized by direct rather than water-mediated hydrogen bonds from Asn^317^ and close contacts to the cytosine C5 position (Fig. 4, *center right panel*). Finally, the sixth position of the FKH motif is commonly cytosine, although thymine is also possible. In both FOXN1 (position 5) and FKH recognition, this is achieved via hydrogen bonds from Arg^320^ to the complementary guanine or adenine, although in the latter the distance between the guanidinium group and nucleobase is too far to make a bidentate interaction, hence the more relaxed specificity (Fig. 4, *bottom right panel*).

In summary, the interactions that recognize the first, third, and sixth positions of the FKH motif are analogous to at the first, second, and fifth positions, respectively, of the FHL motif, whereas the alternate roamer of Asn^317^ switches from recognizing the adenine at position 4 in the FKH motif to the guanine at position 4 of the FHL motif. Hydrophobic contacts to thymine methyl groups have been found to be important for FKH DNA binding in FOXO3 (13), whereas in the FOXN1 close contacts to the cytosine C5 appear to actively preclude binding of thymine at these positions. One exception to this is the FHL-specific yeast FHL1 protein, which contains a serine instead of asparagine at the position equivalent to Asn^317^. In the absence of a structure, it is difficult to draw firm conclusions, but presumably the serine in FHL1 is able to interact with the guanine in the FHL sequence but not make a bidentate interaction with the adenine in the FKH sequence and may thus provide a more direct means of discrimination.

### The C-terminal region of FOXN1 is required for DNA binding

**Figure 5.**
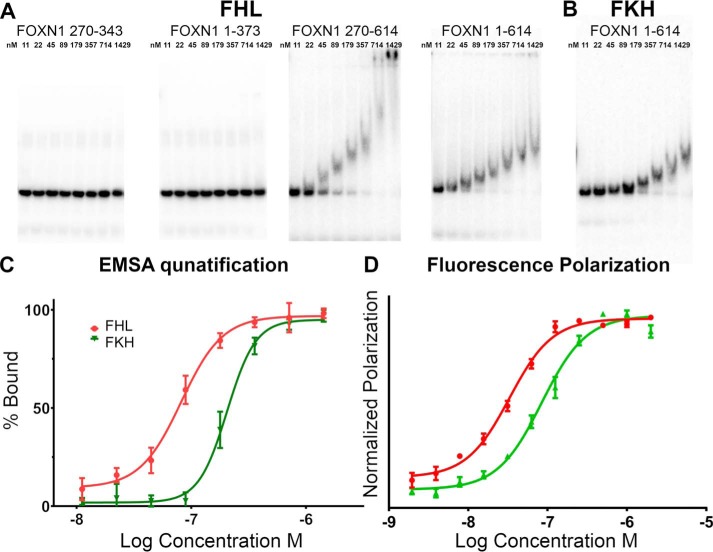
**Measurement of FOXN1 binding to FHL and FKH sequences by electrophoretic mobility shift assay.** *A*, representative gels showing various length FOXN1 constructs binding to a tandem FHL motif. *B*, FOXN1 residues 1–614 binding to an equivalent sequence containing a tandem FKH motif. *C*, quantification of the binding data of FOXN1 1–614 from *A* and *B* and two replicate experiments. *D*, quantification of binding of FOXN1 1–614 to FHL and FKH DNA using fluorescence polarization. *EMSA*, electrophoretic mobility shift assay.

We have performed DNA-binding assays on FOXN1 using electrophoretic mobility shift assays with DNA probes derived from the promoter of the proteasome subunit PSMA7 (a high-confidence FOXN1 site); the strongest binding was seen with a probe containing a tandem arrangement of FHL motifs separated by 10 base pairs. Surprisingly, the forkhead domain alone did not show significant binding activity over the concentration ranges tested (Fig. 5*A*). The addition of the N-terminal region of residues 1–270 did not have any effect on DNA binding, whereas the addition of the C-terminal region (comprising residues 375–608) significantly stimulates binding (Fig. 5*A*). The gel-shift assay shows multiple shifted species with a dose-dependent migration, suggesting that the binding of FOXN1 to DNA may be a more complicated interaction than a simple 1:1 binding event. Nevertheless in the absence of sufficient data with which to fit a more complex model, we were able to quantify from the data an apparent dissociation constant of 82 ± 8 nm (Fig. 5*C*).

As is the case for other FOX family members, regions other than the FH domain of FOXN1 are poorly conserved, whether comparing it with other FOX family members or other metazoan FOXN1 genes. Looking at the sequence in the C terminus of FOXN1, there are no indications of other ordered domains or DNA binding features; moreover, there is a strong prediction of disorder (overall disorder propensity score of 0.74 compared with 0.16 for the FH domain, as calculated using the PONDR server) and a unusually high proline content (55 of 282 residues, or 19.5%), which is generally unfavorable for the formation of distinct secondary structures. We therefore suggest that the C terminus most likely affects the DNA binding by indirect mechanisms, perhaps influencing the oligomeric state of the protein. This would increase the apparent affinity through avidity effects because there is a tandem FHL motif in the probe used. In agreement with this notion, constructs containing the C terminus appeared to behave as multimers in gel filtration, although the likely disordered nature of the C terminus would complicate this analysis and its interpretation.

### Structural determinants of FHL binding

A comprehensive analysis of FOX family protein-binding specificities established three distinct subgroups of DNA specificities (9). First, the FKH-specific, that comprise by far the largest and most varied group (indicating a FKH-specific ancestral origin). Second, the small number of bispecific proteins such as human FOXM1 and FOXN2/3, and third is the FHL-specific groups that include human FOXN1/4 and fungal Fox3. We have decided to test quantitatively the specificity of FOXN1 using a modified version of the high-affinity PSMA7 probe in which the FHL sequences have been changed to FKH consensus sequences with the same relative spacing. Surprisingly the FKH sequence does show some binding activity because the FKH sequence is bound ∼2.5-fold less tightly than the FHL sequence in both the gel shift and fluorescence polarization (FP)–based assays (Fig. 5, *B–D*). The absolute binding affinities appear to be tighter in the FP assay (38 ± 3.3 and 85 ± 11 nm for FHL and FKH DNA) than in the gel-based assay (82 ± 8 and 205 ± 21 nm, respectively). In this case the difference appears to be a result of the ionic strength of the buffer used in the assay, but attempts to match the buffer used in the gel shift assay did not give a good signal in the FP assay (Fig. 5, *B–D*). This ratio of affinities is of a similar magnitude to what was recently determined on FOXN3 (17) (60 nm for FKH and 238 nm for FHL sequences). Given the fact that FOXN3 has been shown to recognize both motifs in cells, we have decided to reanalyze our ChIP-seq data looking specifically for enrichment of the FKH sequence. We had previously identified the GACGC FHL motif as being strongly over-represented (*p* < 0.0001) from a ChIP-seq analysis of Foxn1wt/wt mouse TEC nuclear extracts (5). In contrast, the FKH motif was significantly depleted near FOXN1 ChIP-seq peaks relative to a background of promoter and enhancer regions (*p* < 0.0001). The finding of underenrichment of the FKH motif is surprising given that our *in vitro* measurements of DNA-binding affinity showed only moderate reduction of 2.5-fold. This effect is likely to be driven by chromatin accessibility of potential binding sites; FHL motifs were enriched (*p* < 0.0001), and FKH motifs were depleted (*p* < 0.0001) within ATAC-seq peaks in thymic epithelial cells.

**Figure 6.**
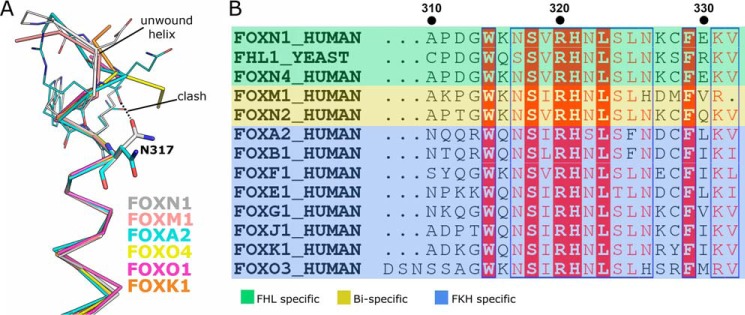
**Sequence determinants of FHL- *versus* FKH-specific binding.** *A*, structural alignment of the N-terminal regions of the recognition helix with the alternate rotamers of Asn^317^ shown. The extended helix present in a subset of the FKH DNA complex structures would prevent the alternate roamer caused by steric clashes. *B*, multiple sequence alignment of a subset of the FOX family proteins from humans and yeast. The specificities of the various proteins were either measured directly or inferred from the phylogenetic analysis in Ref. 17.

As detailed above, one requirement for FHL binding in FOXN1 is the alternative rotamer of Asn^317^. Looking at the surrounding context of this residue in both classes of structures, a clear difference can be seen in the conformation of the N terminus of the recognition helix. Specifically, some of the FKH-specific proteins have an additional turn of the α-helix with a hydrogen bond formed between the main chain amide of the equivalent to Asn^317^ and the carbonyl of the −4 residue. This contact appears to prevent the alternate rotamer of the asparagine caused by a steric clash (Figs. 3*A* and 6*A*). Looking at the sequences in this region, a pattern can be recognized in which the FHL-specific proteins have a proline followed by a negatively charged aspartate and a glycine with the general motif PDGW (Fig. 6*B*); this is the case for the closely related human FOXN1 and FOXN4 proteins as well as the more distantly related yeast FHL1, which is thought to belong to a separate evolutionary clade that evolved FHL-specific activity independently (9). The glycine residue is situated at the helix to coil transition immediately above Asn^317^ and occupies a region of the Ramachandran plot specific to glycine. The negatively charged aspartate is shifted significantly (3.5 Å) away from the equivalent residue in the FKH-binding structures and would have appeared to make unfavorable charge interactions with the DNA backbone if placed in this position. In contrast, the FKH-binding family members generally have either a glutamine or a positively charged residue at this position, which in the structures of FOXK1 (11) and FOXA2 (10) make polar contacts to nearby DNA backbone phosphates, presumably further stabilizing the extended α-helical conformation. We have attempted to test this requirement directly in FOXN1 by mutating the glycine and the preceding motif to representative sequences from FKH specific and bispecific variants. Unfortunately, we were unable to express and purify these chimeric proteins in sufficient quantities for any activity assays.

Additional elements outside of the recognition helix have also been found to influence the specificity DNA recognition of FOX family members. It was recently shown that swapping the wings of FOXJ1 onto FOXN3 reduced the binding to FHL but not FKH DNA sequences in a protein-binding microarray (17). A significant part of this effect can be attributed to a six-residue stretch of amino acids corresponding to residues ^199^LIQALK^204^ in FOXN3 and residues ^356^MQEELQ^362^ of FOXN1. In both FOXN1 and FOXN3 structures, this region is part of an α-helix. We suggest that a general disruption of the secondary structure is responsible for this effect because the FOXJ3 sequence contains two helix-breaking proline residues (^162^VLPTRP^168^) and that an equivalent substitution between FOXN1 and FOXN3 would be unlikely to have a similar effect.

The features that influence the preference in bispecific FOX family members are thus less well-defined, and given the fact that FOXN1 contains moderate FKH binding affinity *in vitro*, there may not be a clear distinction between FHL specific and bi-specific members but rather more of a continuum of specificities. We suggest that although a glycine residue at the equivalent of position 314 in FOXN1 appears to be a requirement for FHL binding, it may not be sufficient to confer this activity, and the preference for FHL over FKH is probably influenced by contacts to the DNA backbone, some of which may come from regions not present in the crystal structures, such as the disordered wings or even contacts from other regions of the protein such as the FOXN1 C terminus. Possible altered binding preferences were recently reported for several wing 2 mutations and C-terminal truncations as well as a helix 3 mutation in FOXA1, which normally binds to FKH motifs (21, 22). Comparing the FHL-bound structures of FOXN1 (moderate preference *in vitro* for FHL) and FOXN3 (moderate preference *in vitro* for FKH) may give insights into how this is achieved. Overall, the structures are very similar (1.1 Å RMSD), with both the conformation of the FHL DNA and the contacts that mediate DNA recognition being conserved (Fig. S1). Only minor differences can be observed in contacts to the DNA backbone, and with the exception of possible differences in the wing1 regions (which are partially disordered in both structures), the only significant difference is an additional water-mediated contact (via Ser^277^) to the DNA backbone in the FOXN1 DNA structure and an additional hydrogen bond to the DNA backbone via Lys^138^ in FOXN3 (Fig. S1). Interestingly these interactions appear to be exclusive to the FHL and FKH DNA conformations, respectively.

### Affinity of FOXN1 for normal and methylated DNA

**Figure 7.**
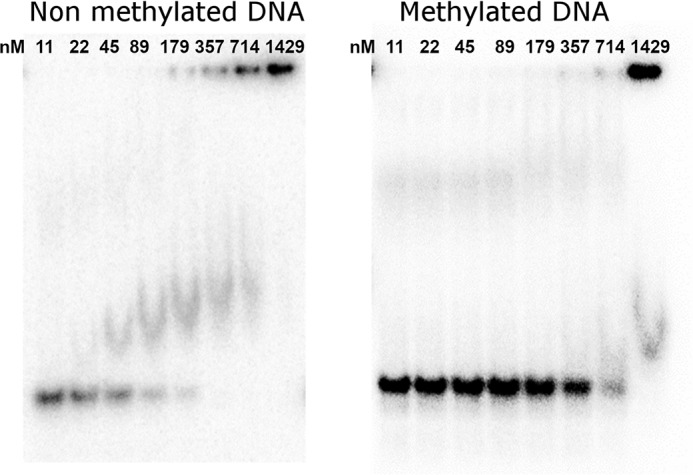
**Electrophoretic mobility shift assay showing FOXN1 DNA-binding activity on consensus sequences containing a 5′ methylcytosine within the FHL consensus motif.**

One further prediction to emerge from the FOXN1 DNA complex structure is that methylation of the CpG within the FHL motif may significantly reduce FOXN1 binding. We have tested this hypothesis using DNA containing 5-methylcytosine at both strands of the CpG sites within the FHL motifs (Fig. 7). This DNA was bound significantly less tightly than the nonmethylated version, with shifts occurring only at the highest protein concentrations where there is also a significant amount of material stuck in wells. This difference in affinity may target FOXN1 binding to CpG islands, regions of nonmethylated DNA that are known to occupy the promoter regions of most human genes (26). Indeed, analysis of the PSMA7 promoter reveals a very strong prediction for a CpG island (>1000 nucleotides with a GC content of >70% and a ratio of observed to expected CpG dinucleotides of ∼1.0).

We tested this model genome-wide using FOXN1 ChIP-seq binding data generated in mice (5). After controlling for the presence of enhancer elements, there was a significant enrichment of FOXN1 binding to FOXN1 recognition motifs within CpG islands (4.2-fold, *p* < 0.0001) but none for FOXN1 motifs located outside of CpG islands (0.9-fold, *p* = 0.1). This provides support for the above model in which FOXN1 binds to its cognate motifs in the context of a CpG island.

Thus there is the distinct possibility that FOXN1 may be under epigenetic regulation, with changes in the methylation of promoter sequences regulating FOXN1 binding. Global DNA methylation patterns are known to change as a function of age (23), with the general pattern of genome-wide hypomethylation and promoter-specific hypermethylation (24). The consequent alteration could lead to a gradual loss of FOXN1 binding to DNA and may thus be a contributing factor to the phenomenon of thymic involution and thus immunosenescence.

## Experimental procedures

### Cloning, overexpression, and purification

FOXN1 constructs corresponding to the FH domain (residues 270–366) and the full-length domain (residues 1–648) were cloned in the vector pNIC28-Bsa4 using ligation-independent cloning and transformed into *Escherichia coli* BL21(DE3)-R3-pRARE2 cells for overexpression (25). The cells were grown at 37 °C in TB medium supplemented with 50 μg/ml kanamycin until an optical density of 2–3, induced by the addition of 0.3 mm isopropyl β-d-thiogalactopyranoside, and incubated overnight at 18 °C. The cells were harvested by centrifugation. For purification, the cell pellets were thawed and resuspended in buffer A (50 mm HEPES, pH 7.5, 500 mm NaCl, 5% glycerol, 10 mm imidazole, 0.5 mm tris(2-carboxyethyl)phosphine (TCEP), with the addition of 1× protease inhibitor set VII (Merck). The cells were lysed by sonication, and cell debris was pelleted by centrifugation. The lysates were loaded on to a nickel–Sepharose IMAC gravity flow column (GE Healthcare), washed with 2 column volumes of wash buffer (buffer A supplemented with 45 mm imidazole), and eluted with 300 mm imidazole in buffer A. The purification tag was cleaved with the addition of 1:20 mass ratio of His-tagged TEV protease during overnight dialysis into buffer A. TEV was removed by IMAC column rebinding, and final protein purification was performed by size-exclusion chromatography using a HiLoad 16/60 Superdex s75 column in buffer A. Protein concentrations were determined by measurement at 280 nm (Nanodrop) using the calculated molecular mass and extinction coefficients. Yields of the FH domain were ∼10 mg/liter, whereas the full-length FOXN1 was expressed at a significantly lower level 0.1 mg/liter. Protein masses were checked by LC/ESI-TOF MS, which discovered the intact mass of the full-length construct of 65,540 Da corresponding to a single truncation at residue 614 in the C terminus. This construct is therefore referred to as 1–614 in the main text.

### Crystallization and structure determination

For crystallization the forkhead domain construct was concentrated to 10 mg/ml using a 10,000 molecular weight cutoff centrifugal concentrator and buffer exchanged to 10 mm HEPES, pH 7.5, 250 mm NaCl, 0.5 mm TCEP. DNA was prepared by mixing the oligonucleotides 5′-GGTGGCGTCTTCA and 5′-TGAAGACGCCACC in a 1:1 ratio at a concentration of 500 μm, heating for 5 min at 94 °C, and letting cool slowly on a heat block. DNA and protein were mixed in a 1.2:1 molar ratio with final protein concentration of 5 mg/ml. The protein–DNA complex crystals grew from conditions containing 8% PEG 4000, 0.1 m acetate, pH 4.5, and the DNA-free crystals grew from conditions containing 10% ethylene glycol, 0.25 m potassium citrate tribasic, 32% PEG 3350. Both crystals were cryoprotected by transferring to a solution of mother liquor supplemented with 25% ethylene glycol and flash-cooled in liquid nitrogen.

The data were collected at Diamond Light Source Beamline I04 (DNA complex) and I03 (DNA-free). Diffraction data were processed with the programs DIALS (26) (DNA complex) and XDS (27) (DNA-free), and the structures were solved by molecular replacement using the program PHASER (28) with the FOXK1–DNA complex (11) structure as a starting model. Model building and real space refinement were performed in COOT (29), and the structures were refined using PHENIX REFINE (30). A summary of the data collection and refinement statistics is shown in Table 1.

### Electrophoretic mobility shift DNA-binding assays

DNA binding was measured using an electrophoretic mobility shift assay. The probes consisted of the following oligonucleotide sequences annealed to their complementary strands (the FOXN1 consensus sites are in bold): 5′-GCAGCA**GACGC**AACAGAGCGA**GACGC**CAGGG (FHL), 5′-GCAGCAGAC^ME^GCAACAGAGCGAGAC^ME^GCCAGGG (methylated FHL), and 5′-AGC**ATAAAC**AACAGAGCG**ATAAAC**CAGG (FKH). The FHL sequence is derived from the mouse PSMA7 gene (chr2:180042455–180042474); the FKH oligonucleotide has a replacement of the two FHL motifs with FKH motifs. Radiolabeled dsDNA probes were prepared by incubating the forward strand oligonucleotides for 2 h at 37 °C with T4 polynucleotide kinase in the presence of [γ-^32^P]ATP. Complementary (nonradiolabeled) oligonucleotides were added in a 2-fold excess, and the mixture was heated to 95 °C and allowed to cool slowly to room temperature. The dsDNA probes were purified on a Bio-Rad P6 micro-biospin column equilibrated in 10 mm Tris, pH 7.5, 50 mm NaCl. Electrophoretic mobility shift assays were performed by incubating radiolabeled probe (at a concentrations ranging between 0.1 and 5 nm depending on the age of the probe) with a 2-fold serial dilution of FOXN1 constructs. The final reaction volume was 7 μl (5 μl of protein stock, + 2 μl of probe) with a final buffer containing 50 mm Tris-HCl, pH 7.5, 25 mm NaCl, 0.5 mm EDTA, 0.1% Tween 20, 2 mm DTT, and 5% glycerol. The reactions were incubated for 30 min at room temperature, and 4 μl of each reaction was loaded on to a 12% native PAGE in Tris–borate–EDTA buffer. The gels were run at 170 V for 70 min on ice. The gels were dried and visualized using phosphorimaging, and quantitation was performed using quantity one-dimensional analysis software (Bio-Rad) measuring free DNA and bound DNA bands. Binding data from three independent measurements were plotted as means ± S.D., and apparent dissociation constants were calculated using a sigmoidal four parameter logistic (*Y* = bottom + (top − bottom)/(1 + 10 ((LogEC50 − *X*)*HillSlope)) nonlinear regression model in PRISM (GraphPad).

### Fluorescence polarization DNA-binding assays

The oligonucleotides 5′-GCAGCAGACGCAACAGAGCGAGACGCCAGGG and 5′-GCAGCATAAACAACAGAGCGATAAACCAGGG were purchased with a 3′ FITC label (Eurofins) and were resuspended to 100 μm in 10 mm HEPES, pH 7.5. The probes were mixed with complementary DNA and heated to 95 °C in a heat block before being allowed to cool slowly to room temperature over 2 h. Probes were used at a final concentration of 10 nm, and assays (30-μl final volume) were performed in 384-well plates in a buffer containing 10 mm HEPES, pH 7.5, 50 mm NaCl, 1 mm TCEP. Serial dilutions of FOXN1 were measured in a PHERAstar plate reader (BMG Labtech) using a 485/520/520-nm filter. Kinetic constants were calculated from binding curves using a four-parameter logarithmic binding equation using the program PRISM (GraphPad).

### FOXN1 ChIP-seq enrichment analysis

FOXN1 ChIP-seq data were derived from Ref. 5. The location of CpG islands was obtained from the University of California, Santa Cruz Genome Browser (mm10 genome build). Enrichment was assessed using GAT with 10,000 permutations and a background determined by H3K27ac peaks in cortical thymic epithelial cells (31). Motif analysis was performed in PScanChIP (version 1.3) using a mixed promoter and enhancer background (32).

## Author contributions

J. A. N. and H. A. conceptualization; J. A. N., G. A. H., and O. G. supervision; J. A. N., H. A., A. E. G., I. A. R., A. E. H., and O. G. investigation; J. A. N. and H. A. writing-original draft; J. A. N., A. E. H., G. A. H., and O. G. writing-review and editing.

## Supplementary Material

Supporting Information

## Footnotes

3 The abbreviations used are:

TEC: thymic epithelial cell
FH or FKH: forkhead
FHL: forkhead-like
RMSD: root-mean-square deviation
FP: fluorescence polarization
TCEP: tris(2-carboxyethyl)phosphine
ESI: electrospray ionization.
