# Supplementary material for: The crystal structure of human forkhead box N1 in complex with DNA reveals the structural basis for forkhead box family specificity
Source: J Biol Chem. 2019 Dec 30;295(10):2948–58. doi: 10.1074/jbc.RA119.010365 (PMC7062188; doi:10.1074/jbc.RA119.010365)
Supplement: Supporting Information [file supp_295_10_2948__index.html]

The crystal structure of human forkhead box N1 in complex with DNA reveals the structural basis for forkhead box family specificity. — FOXN1 and forkhead DNA binding specificity — The crystal structure of human forkhead box N1 in complex with DNA reveals the structural basis for forkhead box family specificity — FOXN1 and forkhead DNA-binding specificity — Supporting Information 

# The crystal structure of human forkhead box N1 in complex with DNA reveals the structural basis for forkhead box family specificity

## Supporting Information

- Supporting Information - Figure S1
