# Supplementary material for: The crystal structure of human forkhead box N1 in complex with DNA reveals the structural basis for forkhead box family specificity
Source: J Biol Chem. 2019 Dec 30;295(10):2948–58. doi: 10.1074/jbc.RA119.010365 (PMC7062188; doi:10.1074/jbc.RA119.010365)
Supplement: Supporting Information [file supp_RA119.010365_154772_2_supp_441175_q28w5p.pdf]

**Supplementary Information: The structural basis for forkhead box family specificity revealed by the crystal structure of human FOXN1 in complex with DNA**

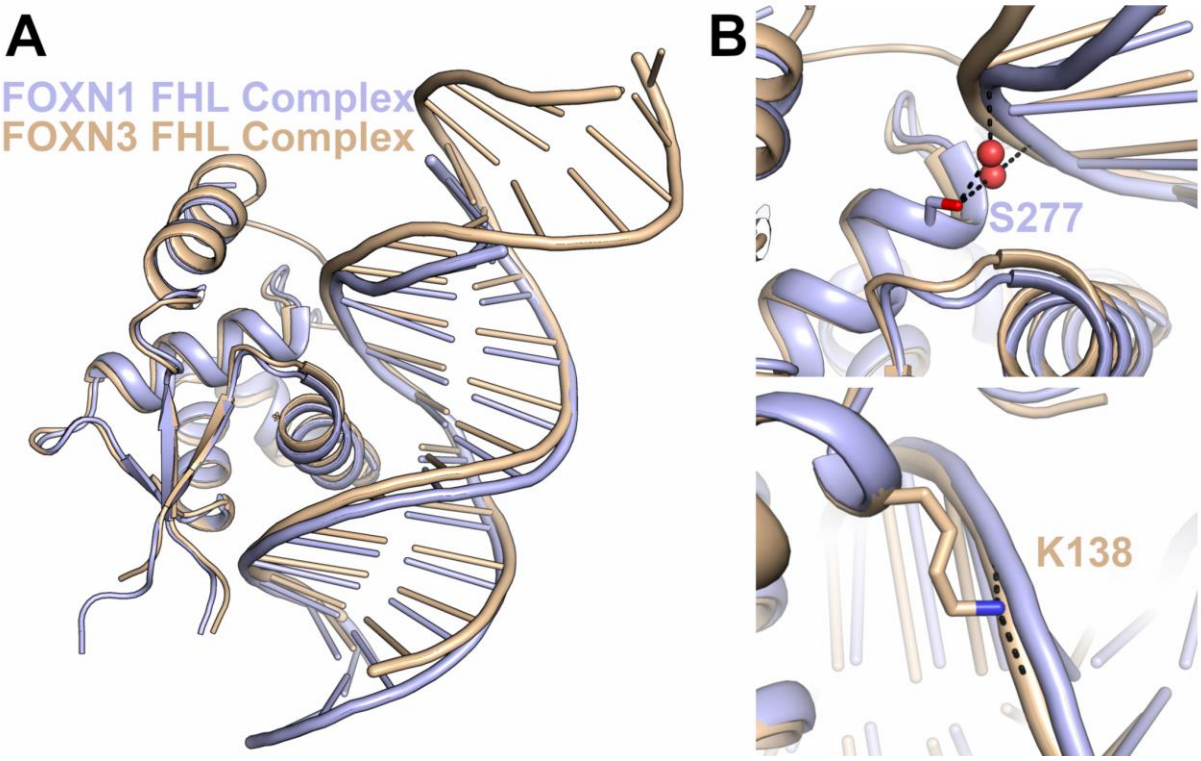


**Figure S1** Comparison between FOXN1 and FOXN3 crystal structures. (**A**) Overall comparison between FOXN1 and FOXN3 both bound to FHL containing DNA. (**B**) Only minor differences in the DNA backbone contacts can be observed, FOXN1 makes an additional water mediated interaction via S277 (upper panel), whilst FOXN3 makes an additional ion pair via K138.
